# Construction Notes

**Published:** 1895-01-05

**Authors:** 


### CONSTRUCTION NOTES.

### New Women's Hospital at Southampton.

The ceremony of opening the new out-patient's department of the Women's Free Hospital has just taken place, the building being erected in Portland Terrace, next to the Royal Victoria Rooms. The plans show a large waiting-room for out-patients, capable of seating some 80 persons. Below this is a well-appointed doctor's consulting-room, and a second waiting-room. Mr. Ingalton Sanders designed the plans for the extension, which were carried out by Mr. A. Warden, of Fitzhugh.

